# Preparation of Silver- and Zinc-Doped Mullite-Based Ceramics Showing Anti-Bacterial Biofilm Properties

**DOI:** 10.3390/molecules16042862

**Published:** 2011-03-31

**Authors:** Suhair Saleh, Mutasem O. Taha, Randa N. Haddadin, Duá Marzooqa, Hamdallah Hodali

**Affiliations:** 1 Department of Pharmaceutical Sciences and Pharmaceutics, Applied Science University, Amman, Jordan; 2 Drug Discovery Unit, Department of Pharmaceutical Sciences, Faculty of Pharmacy, University of Jordan, Amman, Jordan; 3 Department of Pharmaceutics and Pharmaceutical Technology, Faculty of Pharmacy, University of Jordan, Amman, Jordan; 4 Department of Chemistry, Faculty of Sciences, University of Jordan, Amman, Jordan

**Keywords:** mullite, silver, zinc, bacterial attachment, *Pseudomonas aeruginosa*

## Abstract

Zinc- and silver-doped mullite ceramic discs were prepared and tested as potentially resistant materials against bacterial adhesion and biofilm formation. Elemental analysis and X-ray diffraction studies showed that zinc ions were incorporated in the structural framework of the mullite, while silver ions remained outside the mullite crystal lattice, which allowed their slow (0.02 ppm/24 hours) leaching into the surrounding aqueous environment. In agreement with this behavior, silver-doped mullite showed potent resistance against surface attachment of *Pseudomonas aeruginosa*, while on the other hand, zinc-doped mullite failed to stop bacterial attachment.

## 1. Introduction

Biofilms are microbially derived sessile communities characterized by cells embedded in a matrix of extracellular polymeric substances [1,2,3]. Bacterial biofilms are more resistant to antibiotics and biocides compared to planktonic (free) cells [4]. Biofilms occur in medical devices, including contact lenses [4,5] catheters, cardiac valves [4,6], dental implants [7], endotracheal tubes, prosthetic joints, surgical sutures, *etc.* [4] leading to patient suffering, prolonged hospitalization and even death [5,6,8]. 

Several methods have been proposed for keeping surfaces free from bacterial biofilms. These include: (i) addition of a non-adhesive or antimicrobial coating that releases a biocidal agent at the surface. For example, the antimicrobials nisin (a polypeptide bacteriocin) and gramicidin A have been used to protect the surfaces of packaging materials that are in contact with food [2,4]; (ii) introduction of a “shape-shifting” surface or surface-modifying additives. This is exemplified by the use of cationic polymers containing a large number of positively charged nitrogen atoms to treat the skin, oral mucosa and gastrointestinal track and for eradication of micro-organisms on susceptible surfaces [4]; (iii) incorporation of antibacterial metallic surfaces [4]. For example, copper tubing inhibits biofilm formation due to the release of Cu^2+^ ions [9]. The antibacterial properties of silver and zinc have received recent interest because of the wide spread of antibiotic-resistant bacterial strains [10].

Silver is known for its broad-spectrum antimicrobial activity against Gram-positive and Gram-negative bacteria, fungi, protozoa and some viruses [4,11,12]. Silver ions inhibit bacterial enzymes, interfere with electron transport, destabilize bacterial cell walls and membranes and stabilize DNA double helices [12,13,14] which render silver an efficient microbicidal element [15]. In fact, surfaces treated with silver metal, silver oxide or silver chloride were found to inhibit both growth and adherence of *Serratia marcescens*, *Staphylococcus epidermidis*, *Pseudomonas aeruginosa* and *Candida albicans* [16]. By comparison, zinc ions show lower activity as they inhibit nutrient uptake and interfere with proton transfer [14].

Antimicrobial ceramics (ACs) are becoming increasingly important because of their wide range of applications. They consist of a ceramic substrate doped with a certain concentration of diffusible metal ions. AC materials are classified according to the substrate as zeolite ACs, calcium phosphate ACs, amorphous silica ACs, *etc.* [17,18]. 

It has been shown that coatings based on zeolite ceramics containing a combination of Ag and Zn impart significant antimicrobial properties to stainless steel surfaces [14]. Silver zeolite confers antibacterial properties to dental materials, even under anaerobic conditions (e.g., in deep periodontal pockets) [19]. Contact of bacterial cells with silver zeolite leads to transfer of silver into the cells and generation of reactive oxygen species in the cytosol [20]. 

Mullite (3Al_2_O_3_2SiO_2_) is an aluminosilicate–based ceramic of superior physical stability. There is an increasing interest in mullite because of its unique thermal, chemical, dielectric, and optical properties [21]. Recently, selective laser sintering of apatite–mullite with glass–ceramic has been used in bone replacement techniques to yield biofilm-resistant medical prosthetics [22].

The current interest in preparing new materials capable of resisting biofilm formation, combined with the unique physical properties of mullite ceramics, prompted us to envisage new biofilm-resistant ceramics based on mullite-silver and mullite-zinc composites. Nevertheless, silver ions have been reported to interfere and inhibit formation of mullite phase during heating of kaolin [25]. On the other hand, zinc-mullite composites were prepared by robocasting [26].

Such materials should combine the anti-biofilm properties of silver and zinc with the excellent physical robustness of mullite. Furthermore, such systems are completely novel and therefore warrant physical and microbiological investigation.

## 2. Results and Discussion

### 2.1. Preparation and Characterization of Mullite Ceramics

**Figure 1:**
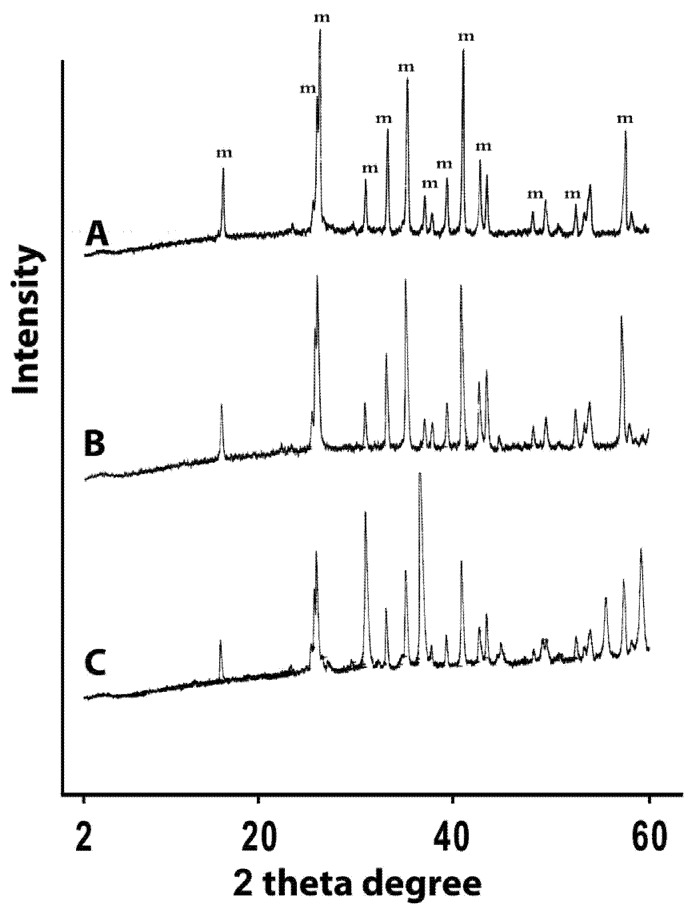
XRD profiles of (A) untreated mullite discs (m = mullite phase), (B) silver-based mullite discs, and (C) zinc-based mullite discs.

Mixing appropriate amounts of Al_2_O_3_ and purified kaolin followed by heating at 900 °C and sintering at 1,450 °C yielded a physically robust ceramic that is comprised mainly of mullite based on the stoichiometric ratio of Al_2_O_3 _to SiO_2_ (Table 1) and the XRD-spectrum (Figure 1). 

**Table 1:** Analysis of different elements in prepared mullite discs.

Analyte	Mullite Blank	Ag-Mullite	Zinc-Mullite	Unit
SiO_2_	24.65	24.84	21.75	%
Al_2_O_3_	70.75	71.73	62.74	%
Zn	78	323	>10000	ppm
Ag	2.2	>100.0	N.A.^a^	ppm
Fe_2_O_3_	0.25	0.26	0.21	%
MgO	0.11	0.09	0.09	%
CaO	0.06	0.04	0.07	%
Na_2_O	0.25	0.19	0.28	%
K_2_O	0.46	0.37	0.41	%
TiO_2_	0.02	0.02	0.02	%
P_2_O_5_	0.04	0.04	0.04	%
MnO	<0.01	<0.01	<0.01	%
Cr_2_O_3_	<0.002	<0.002	<0.002	%
Cu	31	132	130	ppm
Ba	26457	13649	27434	ppm
Ni	<20	<20	<20	ppm
Co	<20	<20	<20	ppm
Sr	709	387	782	ppm
Zr	69	68	60	ppm
Ce	<30	<30	<30	ppm
Y	17	16	14	ppm
Nb	6	7	8	ppm
Sc	2	2	1	ppm
TOT/C	<0.02	<0.02	<0.02	%
TOT/S	<0.02	<0.02	<0.02	%
Sum	99.99	99.74	99.72	%

^a^ N.A. Not analyzed.

However, incorporation of zinc chloride to the preparation procedure significantly altered the stoichiometric ratio of Al_2_O_3_ to SiO_2_. This is clearly apparent from the dramatic reduction of the Al_2_O_3_ percentage in Zn-treated mullite. Similarly, but less drastically, SiO_2_ is also reduced upon incorporation of zinc. On the other hand, doping with silver nitrate seems to have negligible effects on the Al_2_O_3_ to SiO_2_ ratio. These observations suggest that zinc is incorporated into the structural framework of mullite, probably by replacing aluminum and/or silicon, which explains the significant alterations in the composition of the ceramic upon addition of zinc. On the other hand, silver seems to remain outside the chemical framework of mullite. These conclusions are supported by the XRD profiles of the prepared ceramics. Figure 1 shows the XRD profiles of the blank mullite, Zn- and Ag-doped mullite materials with the mullite characteristic peaks [23]. The figure shows clearly identical peak patterns for both the untreated mullite (A) and silver-based mullite (B), suggestive of minimal silver-induced modification on the mullite crystalline structure. However, doping with zinc keeps the same pattern of peaks but alterated their relative intensity, indicating alterations of the Si-to-Al ratio in the structural framework. In conclusion, silver doping seems to preserve the mullite structural framework, in contrast to zinc, which seems to get incorporated into the structural framework of the mullite. This is not unexpected as divalent metal ions are known to get into the structural framework of mullite [24].

### 2.2. Microbiological Evaluation

Table 2 summarizes the anti-bacterial properties of the prepared ceramics. Clearly from the table, zinc-based mullite ceramic failed to resist bacterial attachment and growth of free bacterial (planktonic) cells. On the contrary silver-based mullite caused *ca.* 2 log cycle reduction in planktonic bacteria compared to untreated mullite (blank). Furthermore, silver-based mullite completely inhibited bacterial attachment.

**Table 2:** Results of antibacterial evaluation of prepared ceramic materials.

	Ag-Mullite^c^ (CFU ± SD)^e^		Zn-Mullite^d^ (CFU ± SD)^ e^	
	Blank Discs	Ag-Discs	Blank Discs	Zn-Mullite
****Planktonic cells^a^****	8.06 × 10^7^	2.25 × 10^5^	1.86 × 10^8^	1.88 × 10^8^
	(±5.6 × 10^7^)	(± 2.2 × 10^5^)	(± 6.8 × 10^7^)	(± 6.6 × 10^7^)
****Sessile Cells^b^****	6.03 × 10^4^	0	5.5 × 10^4^	1.10 × 10^5^
	(± 6.2 × 10^4^)		(± 4.9 × 10^4^)	(±1.05 × 10^5^)

^a^ Free cells; ^b^ adhered cells; ^c^ Deionised water was used as growth medium; ^d^ Normal saline was used as growth medium; ^e^ CFU: colony forming unit; SD: standard deviation. Each value represent the average of at least triplicate experiments.

This behavior is not unexpected since zinc ions are deeply entrapped within the structural lattice of mullite, which renders them inaccessible to dissolution into surrounding aqueous environment. On the other hand, silver (which probably exists as silver oxide) seems to be suspended within the ceramic matrix and therefore more available for dissolution into surrounding aqueous environment. This proposition is supported by dissolution studies conducted on silver- and zinc-doped mullites. Silver-doped mullite released 0.02(±0.006) ppm over 24 hours (average of three trials), while zinc-based mullite failed to release any zinc ions in the medium over the same time interval. 

Interestingly, upon assessing the minimal inhibitory concentration (MIC) of silver ions (as silver nitrate) against the same pseudomonal strain used in the current study (ATCC 9027), silver nitrate yielded a MIC value of 2.5 ppm. This finding explains the anti-bacterial effects of the silver-doped mullite, which only caused *ca.* 2 log cycle reductions in planktonic bacterial cells, corresponding to the sub-MIC amounts of silver released over 24 hours (0.02 ppm). Furthermore, despite the significant difference between the determined MIC value and released silver from the ceramic, the amount of leached silver seem enough to prevent bacterial adherence and biofilm formation explaining the complete absence of sessile bacteria on silver-treated mulite discs (Table 2). 

## 3. Experimental

### 3.1 Preparation of Ceramic Discs

Purified kaolin (25.0 g, Riedel-De Haen AG, Germany), γ-aluminum oxide (25.0 g, Merck, Germany) and carboxymethylcellulose (2.0 g, Fluka Chemie AG, Switzerland) were mixed together in a mortar. Subsequently, the mixture was ball-milled (Fritsch Pulverisette 6, Germany) for one hour. The silver-doped sample was prepared by stirring a sample of the ground mixture (10.0 g) in AgNO_3_ aqueous solution (2.5 M, 100 mL, Poch, Poland) at 60 °C for 1.0 hour under dark conditions. Similarly, the zinc-doped sample was prepared by stirring a sample of the ground mixture (10.0 g) in aqueous ZnCl_2_ solution (2.5 M, 100 mL, Panreac, Spain) for 1.0 hour at room temperature. Subsequently, the solid was filtered and dried at 40 °C under vacuum for 2.0 hours. The solid was then re-suspended in de-ionized water (5–7 mL) to form thick paste that was placed in a metal mold (cylindrical aluminum mold with inner diameter of 20.0 mm) and pressed under 50 MPa. The discs were first dried at 110 °C for 2 hours and then heated at 900 °C for 5 hours. Finally they were sintered at 1,450 °C for 6 hours. 

### 3.2. Characterization of Prepared Discs

#### 3.2.1. Powder X-Ray Diffraction

X-ray diffraction (XRD) experiments were performed using Philips 2KW model X-ray diffractometer with monochromated Cu-Kα radiation (λ = 1.5418 Å) and a scan rate of 2° /min.

#### 3.2.2. Elemental Analyses

Elemental analyses of prepared ceramic discs were done using inductively coupled plasma emission spectrometry (ICP-ES) by ACME Labs (www.acmelab.com, Vancouver, BC, Canada). 

#### 3.2.3. Metal Leaching Studies

The concentrations of released metal ions (silver or zinc) were determined via flame atomic absorption spectrometry using a Varian SpectrAA-250 Plus spectrophotometer (Varian, Australia) at emission lines of 328.1 nm and 213.9 nm for silver and zinc, respectively. Five analytical grade standard solutions of silver nitrate (in deionized water) or zinc sulfate heptahydrate (in normal saline) were prepared to construct appropriate calibration curves at: 0.025, 0.05, 0.10, 0.50 and 1.00 ppm (concentration as silver or zinc, respectively). The concentrations of released silver or zinc were calculated from the corresponding calibration curves.

Silver- or zinc-containing ceramic discs were soaked in deionized water (30 mL) or normal saline (0.9% w/v NaCl), respectively, over 24 hours. Subsequently, the dissolution media were collected and analyzed using flame atomic absorption spectrometry. The experiments were repeated three times using three separate ceramic discs of each type (silver- or zinc-based). Deionized water and normal saline solutions were employed as blanks for released silver and zinc, respectively. Each solution was measured in triplicate. 

### 3.3. Antibacterial Studies

#### 3.3.1. Bacterial Strain

*Pseudomonas aeruginosa* (ATCC 9027) strain was used in this study. Culture was maintained on Tryptone Soya Agar (TSA) at 4 °C. To prepare a working culture for attachment experiment, tryptone soya broth (TSB) was inoculated with full loop of culture and kept in shaker incubator at 100 rpm, at 37 °C overnight. A suspension equivalent to 0.5 McFarland (10^8^ cfumL^−1^) was diluted with sterile normal saline (0.9% NaCl) or sterile deionized water to prepare a working solution of 10^6^ cfumL^−1^. 

#### 3.3.2. Media and Chemicals

Tryptone Soya Agar and tryptone soya broth (TSB), sodium chloride, silver nitrate and zinc sulfate heptahydrate were purchased from BDH Laboratory Supplies, Poole, England.

#### 3.3.3. Preparation of Ceramic Discs for Antibacterial Experiments

Ceramic discs were disinfected by soaking in 60% isopropanol for 30 minutes, then they were dried at 60 °C for 30 minutes. Subsequently, they were washed with sterile water (30 mL) and soaked in fresh sterile water (50 mL) overnight to ensure complete removal of isopropanol. Later, the discs were conditioned by incubation in corresponding growth media (50 mL) at 37 °C over 24 hours. Normal saline was used as growth medium for experiments involving zinc discs, while deionised water was used for experiments involving silver discs. Control experiments were performed using blank unloaded discs. 

#### 3.3.4. Evaluation of Discs Resistance against Bacterial Biofilm Formation

*Pseudomonas aeruginosa* were grown overnight in TSB on a shaker-incubator at 37 °C and adjusted to 0.5 McFarland. Subsequently, aliquots of the resulting bacterial suspension were transferred into 50 mL sterile polypropylene centrifuge tubes (BD Falcon, Biosciences). Thereafter, each tube was complemented with corresponding sterile growth media (25 mL, deionised water or normal saline) to give a final viable count of about 10^6^–10^7^ CFU/mL. Subsequently, in each tube a single conditioned ceramic disc was placed and incubated for 24 hr at 37 °C. Then, the discs were removed to measure their anti-adherence activities (see section below). The remaining solutions in tubes were vortexed and aliquots were serially diluted for viable bacterial counting. 

#### 3.3.5. Measuring Anti-adherence Activity of Discs

Discs removed from growth media (see above) were rinsed with three successive 25 mL-volumes of fresh medium to remove loosely attached bacteria. Thereafter, each disc was placed in a tube containing 20 mL fresh medium and sonicated for 10 minutes to remove sessile (adhered) bacteria. Incidentally, sonication over 10 minutes released an average of 0.04 ppm silver from each tested mullite disc. Accordingly, to ensure that the released silver is not responsible for inhibiting bacterial growth in the media collected after sonication (detachment), *i.e.*, which can lead to false negative results, aliquots from the collected media (after sonication) were serially diluted 10, 100, and 1,000 times before viable bacteria counting. This dilution should neutralize any significant anti-bacterial effects due to sonication-induced silver leaching.

#### 3.3.6. Minimal Inhibitory Concentration Determination (MIC)

The minimum inhibitory concentrations (MIC) for zinc and silver were determined by broth tube dilution method. Stock solutions of silver nitrate and zinc sulphate in water were separately prepared and diluted with TSB to have a 20 ppm solution calculated as zinc or silver. Double fold serial dilution in TSB was performed to cover the range from 20 to 0.625 ppm. Aliquot (10 µL) of pre-adjusted overnight microbial culture (0.5 McFarland standards) was used to inoculate each test tube to achieve a final inoculum size of 5 × 10^6^ cfu/mL. After overnight incubation at 30–35 °C, the tubes were inspected for turbidity

## 4. Conclusions

Zinc- and silver-doped mullite ceramic discs were prepared and tested as resistant materials against bacterial adhesion and biofilm formation. Elemental analysis and X-ray diffraction studies showed that zinc ions were incorporated into the framework of the mullite, while silver ions remained outside mullite crystal lattice, which allowed slow leaching into surrounding aqueous environment. In agreement with this behavior, silver-doped mullite illustrated potent resistance against surface attachment of *Pseudomonas aeruginosa*. On the other hand, zinc-doped mullite failed to stop bacterial attachment.
